# Differential Hepatic Metal and Metallothionein Levels in Three Feral Fish Species along a Metal Pollution Gradient

**DOI:** 10.1371/journal.pone.0060805

**Published:** 2013-03-28

**Authors:** Lieven Bervoets, Dries Knapen, Maarten De Jonge, Karen Van Campenhout, Ronny Blust

**Affiliations:** 1 Department of Biology, Systemic Physiological & Ecotoxicological Research, University of Antwerp, Antwerp, Belgium; 2 Laboratory for Veterinary Physiology, Department of Veterinary Sciences, University of Antwerp, Antwerp, Belgium; 3 Department of Environment and Health, Flemish Government, Brussels, Belgium; Federal University of Rio de Janeiro, Brazil

## Abstract

The accumulation of cadmium, copper and zinc and the induction of metallothioneins (MT) in liver of three freshwater fish species was studied. Gudgeon (*Gobio gobio*), roach (*Rutilus rutilus*) and perch (*Perca fluviatilis*) were captured at 6 sampling sites along a cadmium and zinc gradient and one reference site in a tributary of the Scheldt River in Flanders (Belgium).

At each site up to 10 individuals per species were collected and analyzed on their general condition factor (K), hepatosomatic index (HSI) and gonadosomatic index (GSI). From each individual fish the liver was dissected and analyzed on Cd, Cu and Zn and MT-content. Although not all species were present at each site, hepatic Cd and Zn levels generally followed the pollution gradient and highest levels were measured in perch, followed by roach and gudgeon. Nevertheless also an effect of site was observed on this order. MT-levels appeared to be the highest in gudgeon although differences with the other species were not very pronounced and depended on the site. Significant relationships were found between hepatic zinc accumulation and MT levels. For each species the ratio MT_theoretical_/ MT_measured_ was calculated, which gives an indication of the relative capacity to induce MTs and thus immobilize the metals. Perch had the lowest capacity in inducing MTs (highest ratio). Relationships between hepatic metal levels and fish condition indices were absent or very weak.

## Introduction

Despite a decrease in discharges of metals in most West-European countries, including Belgium, several water courses are still historically contaminated with metals [1]–[3], potentially affecting different stages of the aquatic food chain. Fish are interesting species for the evaluation of biological effects of metal pollution under natural circumstances because they might accumulate metals from water, sediment as well as food [4]–[6]. However, accumulation of metals does not necessarily indicates deleterious effects since organisms have possibilities to protect themselves from metal toxicity by increased excretion, differential allocation among organs and by binding the metals intracellularly [7]–[9]. Metallothioneins (MTs) are are low-molecular weight, heat-stable and cysteine-rich proteins involved in the binding and regulation of essential metals such as copperand zinc, and the detoxification of non-essential metals such as cadmium and mercury [10]. The induction of MTs as a response to elevated levels of waterborne and dietary metal exposure, has been frequently used as a biomarker for metal exposure, both under laboratory and field conditions [7], [11]–[20]. Fish species that differ in their feeding strategies and/or detoxification capacities might accumulate metals to a different extent [21]–[24]. Bottom dwelling species such as the gudgeon (*Gobio gobio*) will be exposed to water, sediment and food whereas pelagic species are mainly exposed via water and food.

As a consequence, given the different way of exposure, both metal accumulation and MT-induction might differ among different species [13], [25], which might be responsible for differences in sensitivity to metals [26], [27].

In order to relate possible differences in MT levels to differences in sensitivity multiple effects on fish can be studied. Integrative measures such as condition factor and hepatosomatic index can provide valuable information concerning the overall effect of pollutants on individual fish [28]–[31] and can be related to both metal and MT levels in fish tissues.

The aim of this study was to assess whether different fish species living along a Cd and Zn pollution gradient differentially accumulate metals and induce MTs. Three species were investigated; the bottom dwelling gudgeon (*Gobio gobio*), roach (*Rutilus rutilus*) an invertivorous fish and perch (*Perca fluviatilis*) a piscivorous fish. Metal and MT levels in liver were compared among species and environmental metal concentrations were related to accumulated levels. Furthermore it was investigated whether differences in accumulated metals and induced MTs resulted in different condition. Condition indices that were measured are hepatosomatic index, gonadosomatic index and condition factor.

## Materials and Methods

### Ethics Statement

The study was conducted in accordance to national and international guidelines (directive 2007/526/EC of the European Commission) for the protection of animal welfare. All necessary permits were obtained for the described field studies. Permission to catch fish with electrofishing and to sacrifice a limited number of each species at each location was obtained from the Flemish Government (LNE/Department of Environment, Nature and Energy).

### Study area and sampling design

**Figure 1:**
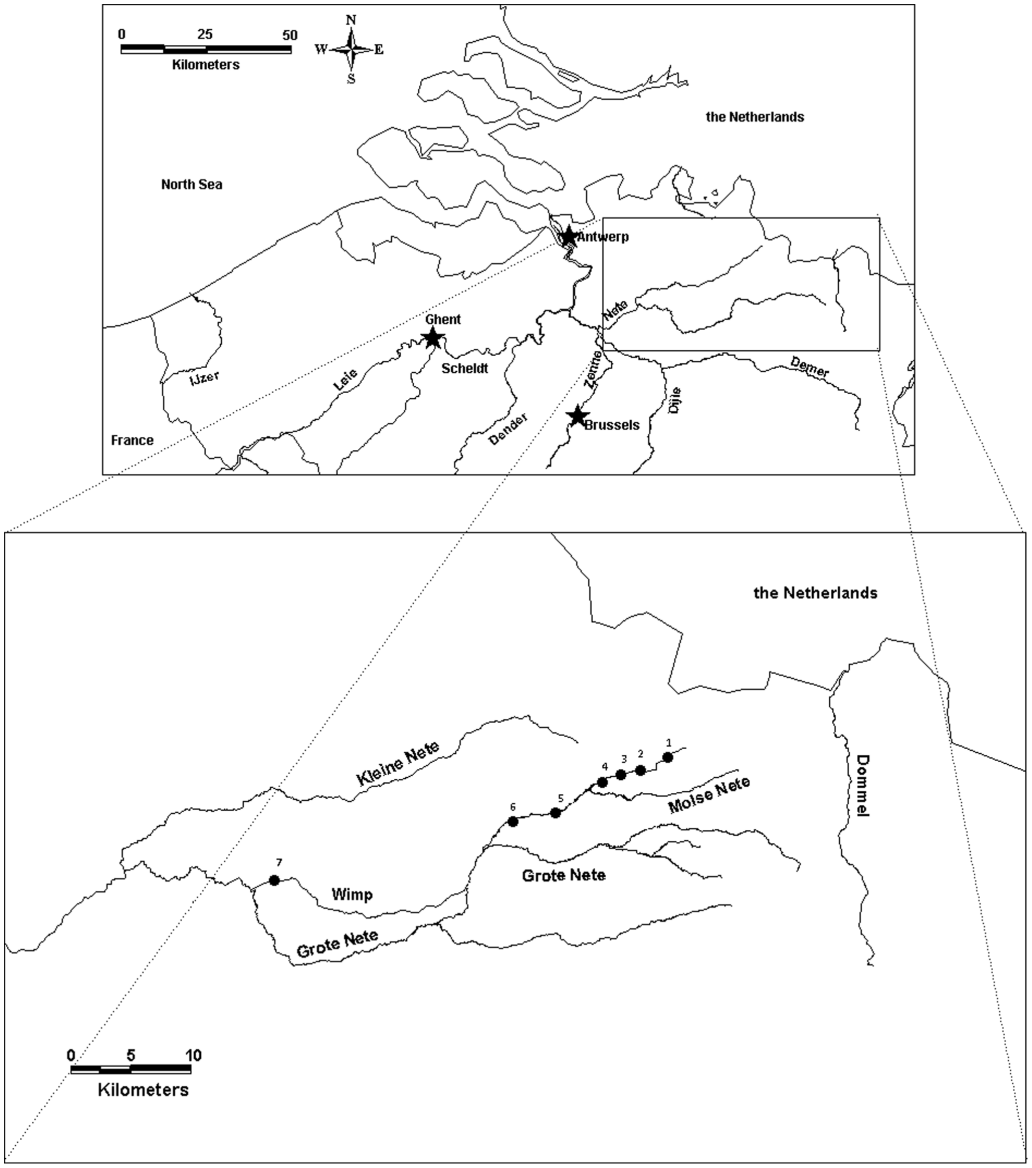
Map of the sampling sites.

Six sampling sites were selected along the River Molse Nete, located in Flanders (Belgium) and belonging to the basin of the River Scheldt. In addition a reference site was sampled (site 7) at the River Wimp, belonging to the same basin (Fig 1). Sampling sites 1–6 are situated along an existing cadmium and zinc concentration gradient with the highest metal concentrations measured at site 2 [28], [31], [32]. Water characteristics such as oxygen level, pH, water hardness and water temperature (4.6 – 6 °C) were within the same range for all sites (table 1).

**Table 1:** Average water quality characteristics at the different sampling sites.

Site	O_2_ mg/l	pH	SO_4_^2-^ mg/l	Cl^-^ mg/l	NO_3_^-^+ NO_2_^-^-N	NH_4_^+^-N mg/l	PO_4_^3—^P mg/l	Cond. µS/cm	Cd µg/l	Cu µg/l	Zn µg/l
1	8.2	7.2	82	51	2.2	0.26	0.05	461	3.9	4.1	712
2	9.3	7.2	85	42	2.0	0.23	0.05	457	62	8.3	5539
3	8.5	7.1	77	39	1.7	0.41	0.08	409	38	6.1	3853
4	8.6	7.2	95	38	1.6	0.35	0.09	409	58	8.1	4864
5	9.3	7.3	61	37	1.5	0.68	0.11	376	17	5.7	1762
6	8.7	7.3	57	36	2.6	0.88	0.16	380	8.4	5.4	922
7	7.6	6.8	60	53	1.6	2.2	0.13	391	< 0.10	3.5	62
CQC	5.5-9.5	6.5–9.0	500	250	NA	*	NA		0.02	24	30
FQC	≥ 5.0	6.5–8.0	250	200	10.0	1.0	0.30	1000	1.0	50	200

CQC: Canadian Quality Criteria for aquatic freshwater life [65] FQC: Flemish Quality Criteria [66] NA: Not Available; * pH-dependent; ND: Not Determined

Water samples were collected in duplicate monthly between November and March 2001–2002 at all sampling stations. To measure the total metal concentrations water samples were acidified with nitric acid (HNO_3_; 69%) to a pH of 2.0 and filtered through a 0.45-µm Millipore (Bedford, MA, USA) membrane filter. All samples were stored in 20-ml polypropylene vials at 4 °C until analysis. Sediments were collected twice, i.e. in December and in February. Samples were taken with a 'Petit Ponar' grab sampler (Wildco cat.no. 1728; 235 cm^2^). At each site a mixed sample was taken, composed of 5 grab samples [33]. Samples were sieved with the site-water using a 500 µm-mesh sieve and stored in 500 ml polyethylene beakers at 4°C. Subsequently, supernatant was decanted carefully to prevent loss of sediment. After decantation the sediment sample was homogenised with a plastic spatula. Prior to extraction the sediment of each sampling station was centrifuged (10,000 *g*) to collect the pore water [34]. The remaining wet sediment was analysed on total metal content: sediments were dried at 60 °C during 48 hours and a mixture of concentrated HNO_3_ and HCl (4∶1) was added. Eventually, samples were put in Teflon bombs and digested in a microwave oven [28], [35].

At all sampling sites fish were caught within one month between December and January by electrofishing, using an Electra catch WFC7 generator producing 150 V. Three successive samplings were conducted at each site (length: ± 100 m). All fish were identified to the species level and counted. Of all specimens, forklength was measured (± 1 mm) and weight determined using a Kern 442.43 balance (± 0.1 g).

From each sampling site up to 8 specimens (if present) of three fish species, i.e. gudgeon (*Gobio gobio*), perch (*Perca fluviatilis*), and roach (*Rutilus rutilus*), were sacrificed using an overdose of the anesthetic ethyl meta-aminobenzoate methanesulfonic acid (MS 222) and liver tissues were collected and weighed (0.001 g) in the field and immediately stored in liquid nitrogen and transported to the lab. In the lab, the liver was homogenized and separated in two parts, one part for determination of metal concentrations stored at –20 °C and one part for metallothionein determination stored at –80 °C. Tissue samples for metal determination were put in acid-washed polypropylene pre-weighted vials and dried for 24 h at 60 °C. Subsequently, the biological material was digested with a mixture of concentrated ultrapure HNO_3_ and hydrogen peroxide (H_2_O_2_; 29%) (3:1; v/v) in a microwave oven and stored until measurement [28].

### Chemical measurements

Water quality characteristics (pH; temperature (°C); conductivity (µS/cm); oxygen (mg/l)) were measured on-site and at each sampling moment with a WTW multiline F/SET-3 field kit. Chloride, sulphate, phosphate, ammonium, nitrate and nitrite were determined using an automated analyser (Skalar: FAS, SA 20/40).

Cadmium, copper, and zinc were measured in the surface water, sediment pore water, bulk sediment and fish tissue samples with either an Inductively Coupled Plasma-Atomic Emission Spectrometer (ICP-AES, Varian Liberty Series II, Victoria, Australia) equipped with a micro concentric groove nebulizer [36] or an Inductively Coupled Plasma-Mass Spectrophotometer (ICP-MS, Varian UltraMass 700, Victoria, Australia).

Concentrations of the metals in the water are expressed in µmol l^−1^, the liver metal concentrations are expressed on a wet weight (wet/wet) basis in nmol g^−1^. The accuracy of the metal determination was verified using certified reference material of the Community Bureau of Reference (EU), i.e. standard for trace elements in river sediment (CRM 320) and mussel tissue (CRM 278). Recoveries were within 10 % of the certified values.

### Metallothionein determination

After thawing, the tissue samples were homogenized using an Ultra-turrax T8 (IKA, Labortechnik, Staufen, Germany) in 3 volumes (v/w) of 10 mM Tris-HCl buffer (pH 7.4) containing 5 mM β-mercaptoethanol (to prevent oxidation), and 0.1 mM phenylmethanesulfonylfluoride (PMSF, protease inhibitor). Tris-(hydroxylmethyl)-aminomethane (Tris), PMSF, and β-mercaptoethanol were obtained from Sigma (Sigma-Aldrich, St. Louis, MO, USA). The samples were centrifuged at 9000 *g* and 4 °C for 10 min. (Eppendorf Centrifuge 5804R, Hamburg, Germany) and ultra centrifuged at 100 000 *g* for 60 min. at 4 °C (Sorval Discovery TM 90 Ultra speed centrifuge, Newton, Connecticut, USA). Aliquots of supernatants (cytosolic fractions) were stored at – 80 °C for a period not longer than 24 hrs before use. Total cytosolic MT concentrations were measured using the cadmium thiomolybdate saturation assay from Klein *et al.* [37].

The general concept of this assay is to remove all MT-bound Cd, Cu, and Zn ions and, subsequently, to saturate the MT molecules with the ^109^Cd isotope, which was quantified using a Minaxi-Autogamma 5530 counter (Canberra Packard, Boston, MA, USA). The excessive ^109^Cd was complexed by Chelex-100 (Merck, Darmstadt, Germany). The MT concentrations were calculated on the basis of a molar ratio of Cd/MT of 7 [38]. It has been shown that this method compares very well with other established techniques such as ELISA or the Ag-saturation method [39].

Measuring MT concentrations using the cadmium thiomolybdate saturation assay of Klein et al. [37] allows the determination of MT binding places including the ones initially occupied by Cu, Cd and Zn. Measured MT concentrations in liver were compared to Cu, Cd and Zn concentrations measured in those tissues. In order to investigate differences in detoxification capacities among the three fish species, the theoretical level of MT needed to bind all the present Cd, Cu and Zn in the liver was calculated for the three species. Based on the specific binding capacity of the thiol groups of MT for the three metals (7 mol Cd, 12 mol Cu and 7 mol Zn/mol MT, Kito et al. [38]) the following equation was used: MT_theoretical_  =  [Cd]tissue/7 + [Cu]tissue/12+[Zn]tissue/7. The ratio MT_theoretical_/MT_measured_ (further referred to as MT_t_/MT_m_) provides an indication of how efficient an organism is inducing MTs after exposure to metals.

### Condition indices

The relative condition factor (K) was calculated according to the formula K  =  W/W’ [40], [41], where the observed weight of an individual fish (W in g) is compared to its expected weight (W’) based on its observed length (L). This expected weight is derived from the length-weight regressions W  =  a L^b^, with *a* and *b* as fitting constants from reference populations of each studied species. K indicates whether an individual of a species is in a better (>1) or worse (<1) condition compared to an average individual of the same length in a reference population.

The Hepatosomatic Index (HIS) and Gonadosomatic Index (GSI) were calculated by comparing the respective organ weights to the total body weight (organ weight/total body weight x 100).

### Statistical analysis

ANOVA (Analysis of Variance) and Duncan’s multiple range tests were used to determine the significance of the differences between the different sampling sites. If the data were not normally distributed or showed heterogeneous variances, they were log transformed to get homogeneous variances and a normal distribution. Multiple linear regressions were used to construct empirical models to relate exposure concentrations to the tissue metal concentrations and the tissue MT concentrations. All statistical analyses were performed using the software package STATISTICA (StatSoft Inc., Tulsa).

## Results

Table 1 summarizes the results of the water quality characteristics from the different sampling sites. General water quality characteristics all met the Flemish and international water quality standards. Total Cd and Zn levels clearly followed a gradient between site 2 and site 7. For copper no pattern was observed. Cd and Zn levels exceeded quality standards at all sites, including site 1 upstream of the pollution source but except the reference site,

Table 2 gives the results of the sediment and pore water analysis. Cadmium and Zn concentrations in the sediment did not follow the same gradient as for the dissolved metals but also exceeded sediment quality standards. Highest levels of both metals were measured more downstream of the pollution source. At the downstream sites increased copper levels were measured although nowhere the quality standards were exceeded.

**Table 2:** Total metal levels in the sediments and the pore water at the different sampling sites.

Site	Cd-sed µg/g dw	Cu-sed µg/g dw	Zn-sed µg/g dw	Cd-pw µg/l	Cu-pw µg/l	Zn-pw µg/l
1	1.88	1.64	89.7	1.23	0.43	26.4
2	19.3	2.56	313	nd	nd	nd
3	35.3	3.15	1431	4.95	1.14	1275
4	51.6	6.78	1625	2.05	1.55	341
5	40.8	18.6	2306	1.43	0.42	95.0
6	51.8	35.7	2634	1.19	1.29	555
7	0.27	9.17	177	0.12	2.80	17.0
CCC				2.2	9.0	120
PEC	3.5	197	315			

Sed: sediment; pw: pore water

CCC: Criterion Continuous Concentration [43] aquatic freshwater life; ND: Not Determined

PEC: Probable Effect Concentrations [42].

For each species fish of comparable size were selected for analysis (total length: roach: 13.9 ± 2.8 cm; gudgeon: 11.1 ± 1.6; perch: 14.0 ± 5.6 cm). Roach could be captured in sufficient numbers at all sites. Gudgeon however, was absent at site 2 and only one specimen was captured at sites 3 and 4. Perch was absent at site 2. In total, metal and MT levels in the liver could be measured in 45 roach, 23 perch and 33 gudgeons captured in the pollution gradient.

**Figure 2:**
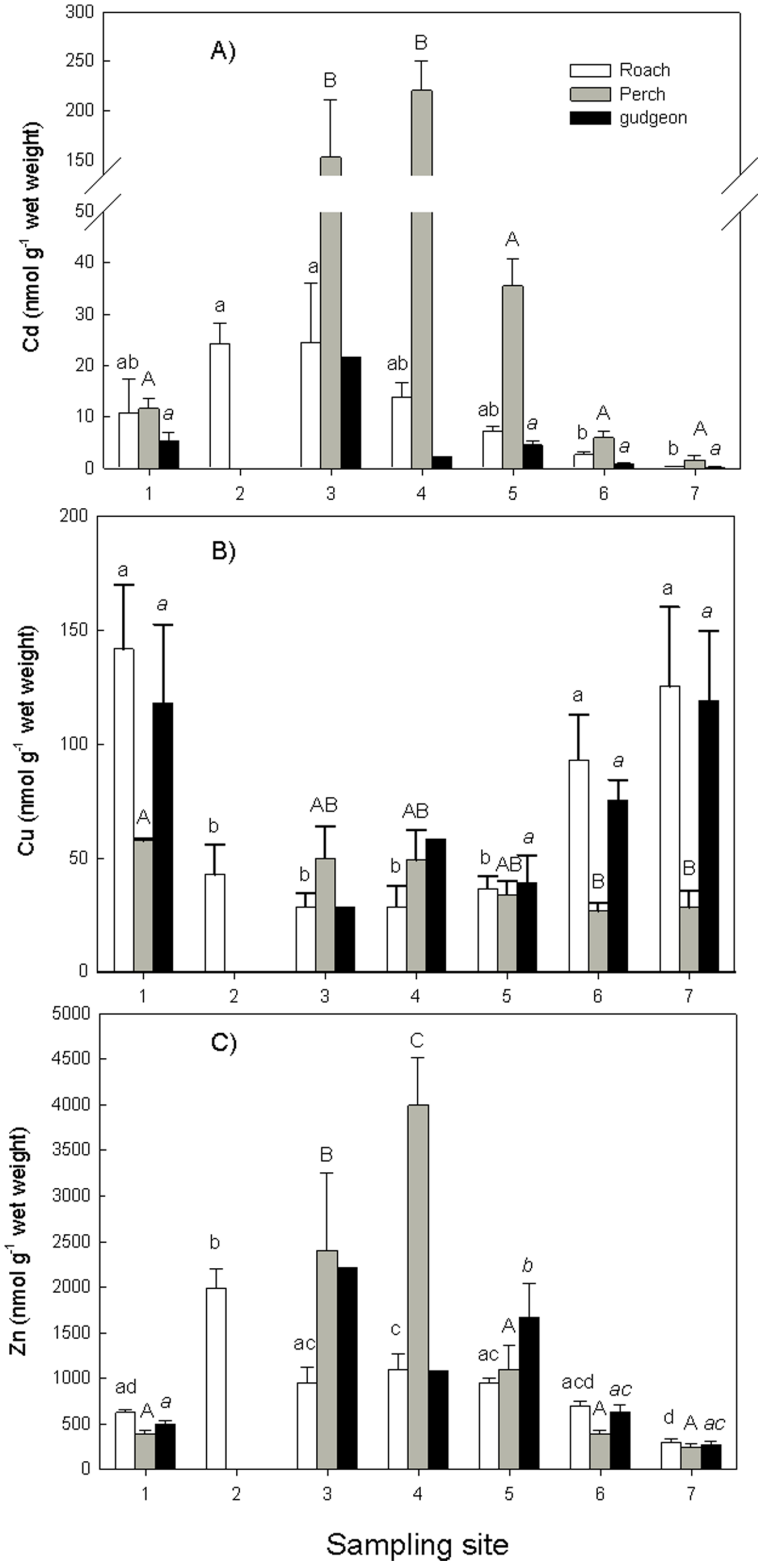
Mean metal concentration (± standard deviation) in liver of the three fish species in µg g^−1^ dry weight. (a) Cd, (b) Cu, (c) Zn. Different letters (A, a or *a* for respectively Roach, Perch and Gudgeon) mean significant differences in hepatic tissue within a species (p < 0.05).

Figure 2 shows the results of the Cd, Cu and Zn concentrations in the liver of the three fish species caught at the different sampling. The average wet weight/dry weight ratios for the liver were 3.63 ± 0.39, 3.85 ± 0.31, 3.05 ± 0.60 for Roach, Perch and Gudgeon respectively. The effect of site and species was analyzed with a two-way ANOVA. Because not all species were present at all sites this two-way ANOVA could be applied only on fish from sites 1, 5, 6 and 7. One-way ANOVA, followed by a Duncan’s multiple range test, was used to compare the levels among the sites per species. For Cd there was a significant effect of site and species and a significant interaction (table 3). Highest cadmium levels were measured in liver of perch at site 5. Considering the species separately, in roach highest hepatic cadmium levels were measured at site 2 and 3, in perch at site 3 and 5 and in gudgeon at site 3 (no perch or gudgeon were caught at site 2). Also for Cu there was a significant effect of site and species but no significant interaction (table 3) with highest levels in roach and gudgeon at the sites 1, 6 and 7. For gudgeon however, no significant differences among sites were observed and also for perch differences were less pronounced with significant lower levels at site 6 and 7 compared to site 1 (Figure 2B). For zinc again there was a significant effect of species and site and a significant interaction (table 3). Significant highest zinc levels were measured in liver of perch at site 3 and 4. Looking at the individual species the pattern found was comparable to cadmium with for roach highest levels at site 2, for perch highest levels at site 4 and for gudgeon highest levels at site 5.

**Table 3:** Two-way analysis of variance for the effect of species and site on hepatic metal levels.

Source of variation	df	Mean of Squares	F_s_
Cadmium			
Species	2	17932	90.7^***^
site	4	8017	40.5^***^
Interaction	8	10695	54.1^***^
Zinc			
Species	2	689305	2.23^ns^
site	4	4287589	13.9^***^
Interaction	8	893124	2.89^**^
Copper			
Species	2	22891	9.10^***^
site	4	15777	6.27^***^
Interaction	8	2744	1.09^ns^

**Figure 3:**
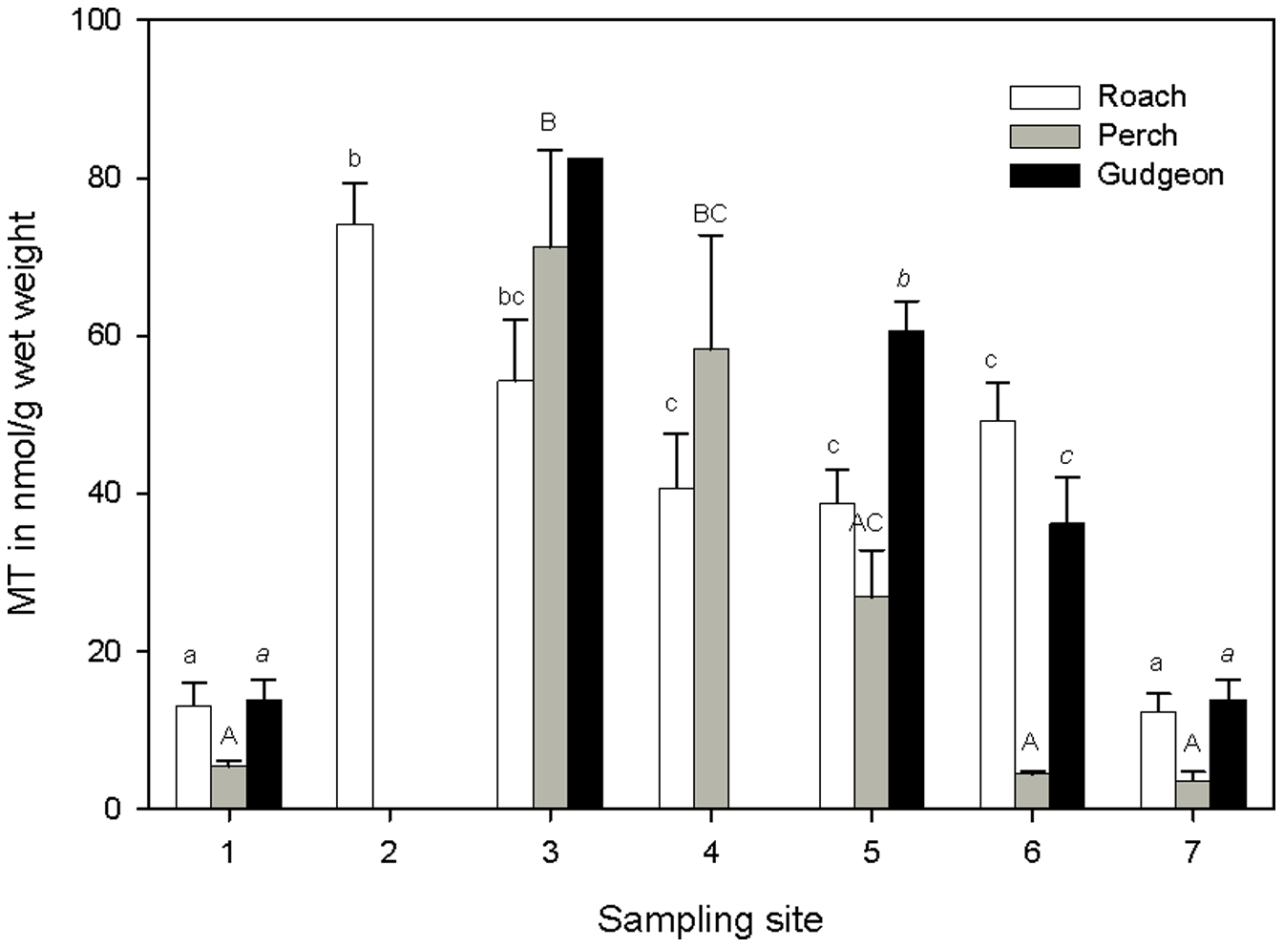
Metallothionein level (MT) in liver of the three fish species (nmol MT g-1 wet weight). Different letters (A, a or *a* for respectively Roach, Perch and Gudgeon) mean significant differences in hepatic tissue within species (p < 0.05).

Figure 3 shows the hepatic MT levels in the 3 species at the different sites. The effect of site and species on MT levels was analyzed with a two-way ANOVA. Because no sufficient numbers of all species could be captured at all sites, this two-way ANOVA could be applied only on fish from sites 1, 5, 6 and 7. Significant effects of site and species and a significant interaction were found (table 4). Significant highest hepatic MT levels were found in gudgeon from site 5. When the MT levels in the individual species were compared among sites, in roach highest levels were found at sites 2 and 3, in perch at 3 and 4 and in gudgeon at site 3, but here only one individual was captured.

**Table 4:** Two-way analysis of variance for the effect of species and site on hepatic Metallothioneine levels.

Source of variation	df	Mean of Squares	F_s_
Species	2	2214	16.5^***^
site	4	3852	28.8^***^
Interaction	8	688	5.14^***^

**Figure 4:**
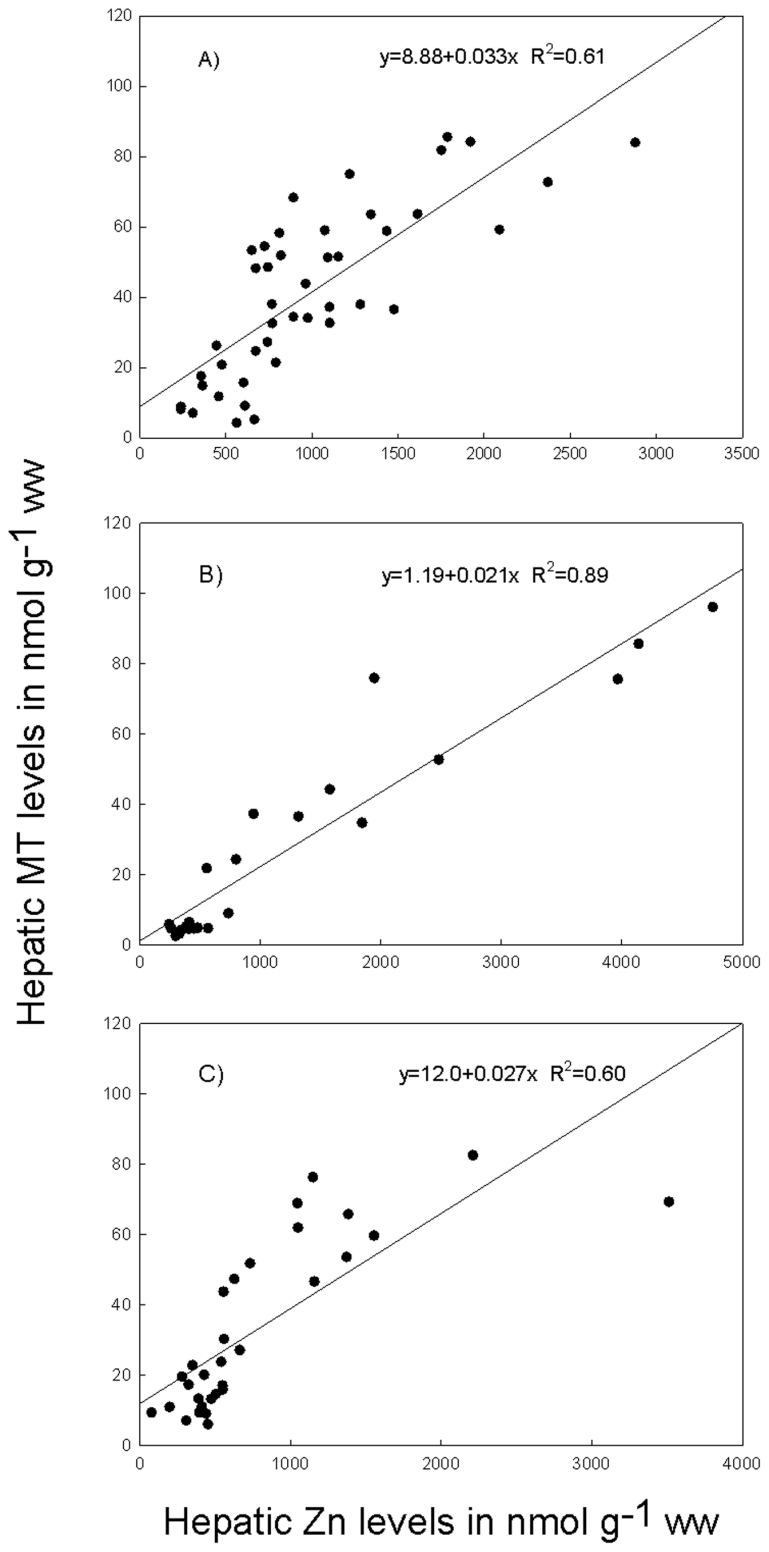
Relationship between the hepatic Zn and MT level in the three fish species. (a) Roach; (b) Perch; (c) Gudgeon.

**Figure 5:**
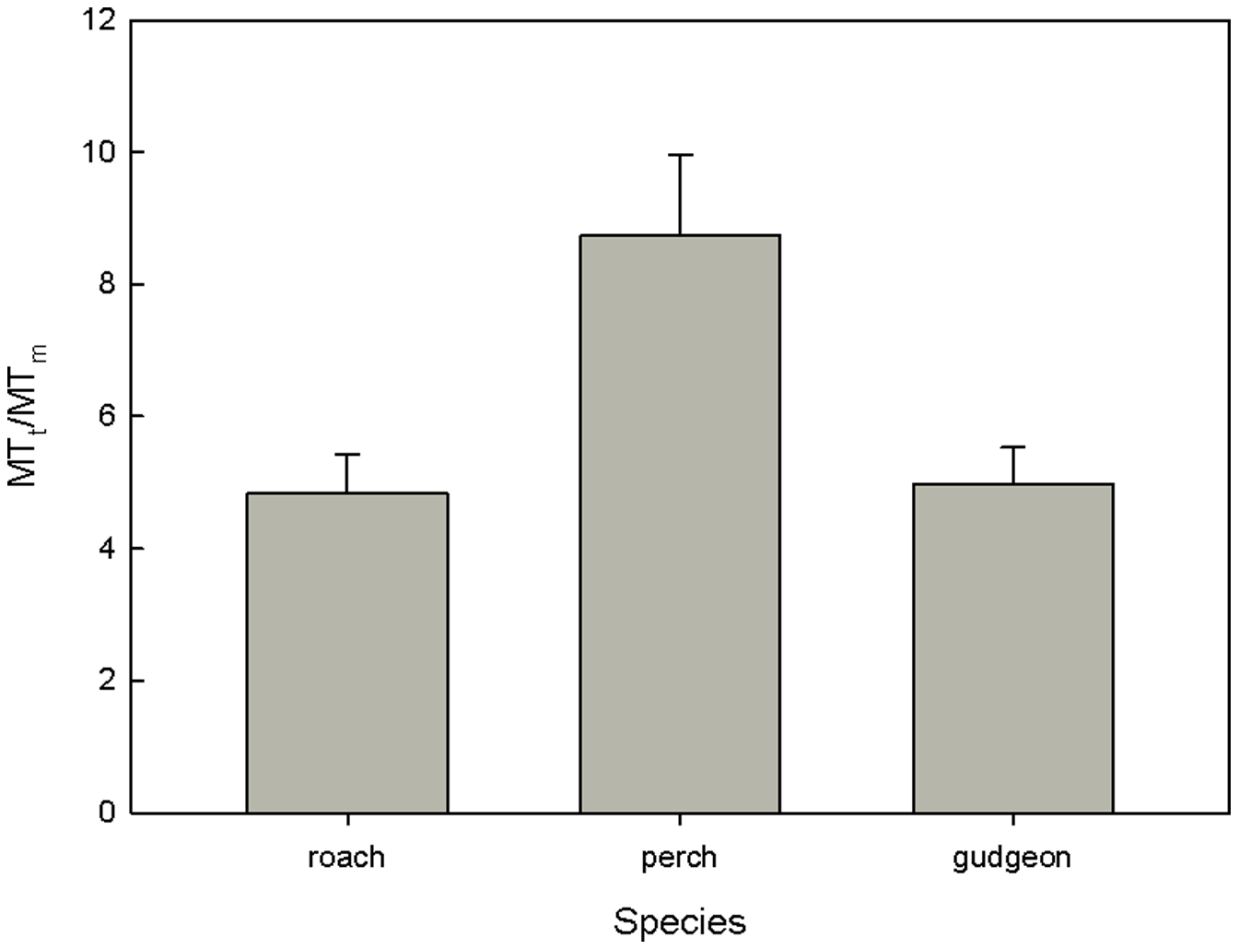
Ratio of theoretical hepatic MT/measured hepatic MT for the three fish species.

Multiple regression analysis linking measured MT concentrations to Cu, Cd and Zn concentrations in liver showed strong relationships for all three species with R^2^ of 0.63; 0.92 and 0.61 for roach, perch and gudgeon respectively. However, the only metal that was significantly contributing in the linear regression to the MT levels was Zn. However, Zn was strongly correlated with Cd and including only Cd in the model also revealed significant relationships with R^2^ between 0.60 and 0.87. Figure 4 illustrates the relationship between hepatic zinc and MT levels for the three species. Figure 5 presents the MT_t_/MT_m_ ratio for the three species. The higher the ratio the lower is the efficiency. Perch proved to induce lower MT levels compared to the two other species (ANOVA, figure 5).

**Figure 6:**
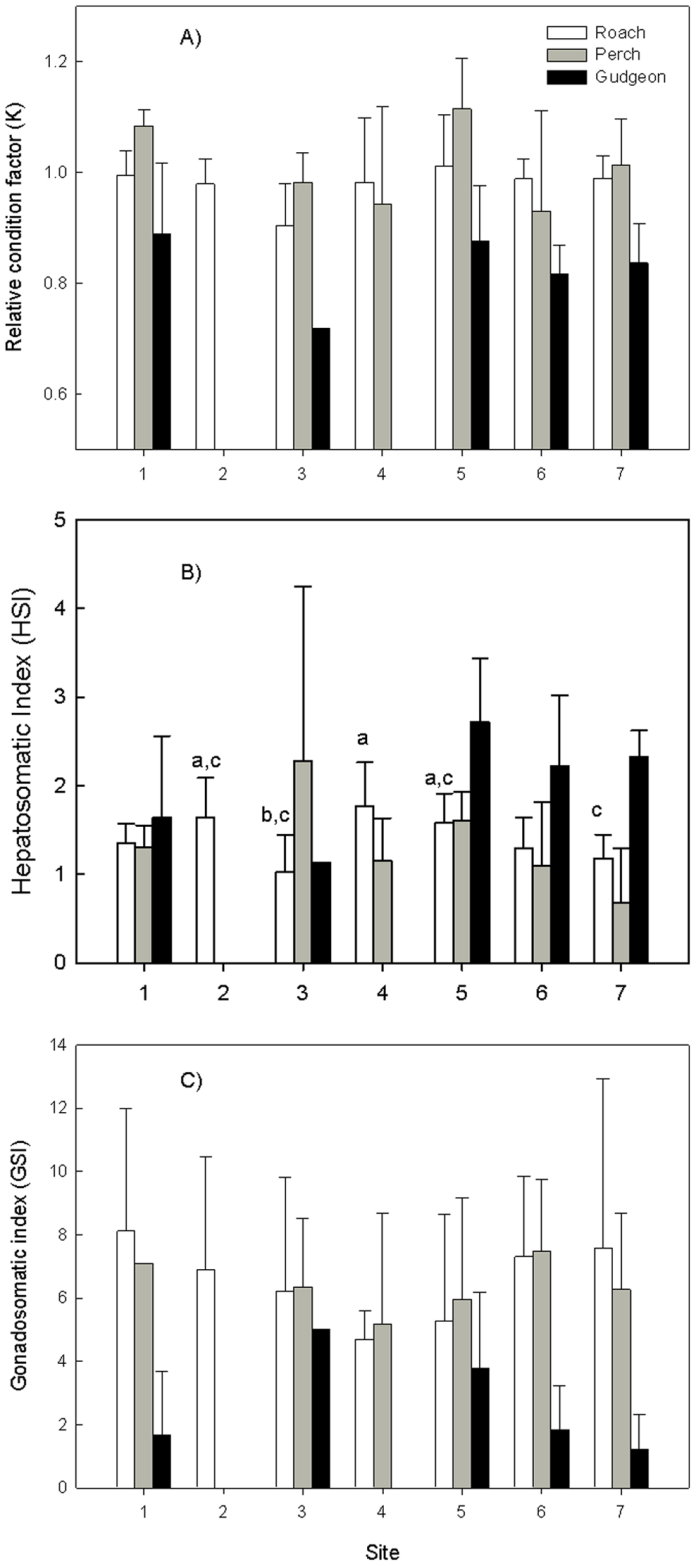
Condition measures for the three fish species (mean ± standard dev.). A) Relative condition factor; B) Hepatosomatic Index; C) Gonadosomatic Index. Different letters mean significant differences within species (p < 0.05)

Figure 6 summarizes the results of the different condition indices measured for the three fish species. Values were tested on significant differences with an ANOVA, followed by a Duncan’s multiple range test. For Roach only significant differences were observed for HSI with lowest levels at site 3 and highest at site 4. From site 4 on HSI decreased more downstream. For Perch only K differed significantly among the sites with highest levels at site 5. For gudgeon no significant differences were found for none of the measured indices among the sites.

With regression analysis the individual K, HSI and GSI were related for each species to the individual hepatic metal concentration. For Roach a significant negative relationship was found between hepatic Cd and K although the R^2^ was only 0.16. For Perch significant but also rather week, negative relationships were found between hepatic Cd and GSI and between hepatic Zn and K (R^2^ of respectively 0.14 and 0.17). Finally for gudgeon a significant negative relationship was found between hepatic Cd and HSI (R^2^ = 0.19).

Relating the MT_t_/MT_m_-ratio to the condition indices revealed only a significant negative relationship with K for perch (R^2^ = 0.17) and with HSI for gudgeon (R^2^ = 0.25).

## Discussion

Cd and Zn in surface water and sediment exceeded the Flemish and International quality standards [42], [43] at all investigated sites, except the reference site. Also compared to other polluted sites the measured environmental levels can be considered as very high [34], [44], [45] and among the highest levels reported before [46]. This was reflected in the hepatic Cd and Zn levels of the three fish species. Hepatic levels in roach ranged from 0.01 to 9.17 µg/g dw for Cd, from 0.62 to 18.4 µg/g dw for Cu and from 15.5 to 185 µg/g dw for Zn. Cadmium and zinc levels measured in fish from the present study were comparable or slightly to much higher than the levels measured in liver from roach and perch from metal polluted sites in literature whereas copper levels were comparable to levels in fish from uncontaminated sites [19], [31], [32], [47]–[50]. For gudgeon only data were found from the same pollution gradient but from another period and levels in the present study were slightly lower [31], [32], [51].

In the present study hepatic Cd and Zn levels were higher in perch than in gudgeon and roach at most of the sites. This was opposite to what was found by Blanchard et al [49], Teil et al. [52] and Szarek-Gwiazda et al [51] who all found equal or lower Cd and Zn levels in perch compared to roach but in their studies environmental levels were lower compared to the present study. Andres et al. [48] however, measured higher levels in roach than in perch at environmental levels comparable to the present study. We cannot find any explanation for these differences in literature. Although in most studies no biomagnification is observed for metals other than Hg [53], in the present study highest levels were measured in the piscivorous fish. A possible reason for the highest levels in perch is that this species could be less capable in eliminating the metals than the other two species.

Differences in hepatic MT levels among the three fish species were site-specific although at most sites MT levels were comparable between roach and gudgeon but much lower in perch. At the reference site average MT levels were 12.4, 3.65 and 13.9 nmol/g ww for roach, perch and gudgeon respectively.

MT levels in roach from the reference site were comparable to levels found in unexposed roach [54], [55] or from a reference site in the same River basin [19]. Studies of hepatic MT levels in gudgeon could only be found from the same river basin [16], [26]. Levels in the present study from the reference site were comparable or slightly lower than the levels found by Van Campenhout et al [16] and Knapen et al. [26] at reference sites. At the contaminated sites MT levels in gudgeon in the present study were slightly to much lower compared to the levels found by Van Campenhout et al [16], which measured MT levels in gudgeon sampled in 1998 from the same contaminated sites. This is probably due to the decreased metal levels between the two study periods [16]. Levels in perch from our study were comparable to the levels found by Hogstrand et al. [56].

For all three species good to very good relationships were found between hepatic zinc and hepatic MT levels (figure 4). Several studies have investigated the effect of pollutants on the condition factor, HSI or GSI [e.g.19,57–59]. In the present study few differences in K, HSI or GSI were found among the sites for the different species.

Few significant and rather weak relationships were found between hepatic metal levels and condition indices. Ozmen et al. [60] found a reduced condition factor (CF) in carp captured at sites polluted with various contaminants including metals. A strong significant inverse relationship between CF and Cd concentration in liver of brown trout was found in a study of Clements and Rees [57]. Reynders et al. [19] found significant differences in CF of caged carp among sites but no significant relationship with accumulated metal. In a study of Bervoets et al. [61] condition factor was significantly related to metal load in the kidney but not in the other tissues. Knapen et al. [31] however, found significant differences in K for gudgeon along the same pollution gradient and reference site. However, lowest K was observed at the reference site and the least contaminated site of the gradient. Other studies did not find any relationships between environmental pollution and condition factor in fish [59], [62]. In the present study the described variation in CF by hepatic metal levels was rather low, which can be attributed to other factors than (metal) pollution such as habitat quality and food availability [63].

A negative but weak relationship was found for gudgeon between hepatic Cd and the hepatosomatic index. This is in contrast to what was found by Ozmen et al. [60] and Bervoets et al. [61] who found a positive relationship between hepatic Cd levels and HSI in carp. However, Pereira et al. [64] measured a decreased HSI in winter flounder (*Pleuronectes americanus*) exposed to high cadmium concentrations for 71 days.

Since MTs are also induced by Hg and Ag, the approach of the MTt/MTm-ratio is only valid in a study area with low levels of those metals. In the present study we Hg and Ag were measured in the livers (data not shown) but levels were not significant among sites and species and generally very low or often even below the detection limit. Moreover, from the database of Flemish Environment Agency (www.vmm.be) it was obvious that concentrations of both metals were low in the pollution gradient.

If we compare the ratios of the theoretical over the actual MT levels perch proved to induce less MTs compared to the two other species. From this observation we would expect that perch is more sensitive to the metal pollution than the other two species. This was however not tested in the present study and relationships between the MTt/MTm-ratio and condition indices were non-significant or only weak relationships were found such as for perch with K and for gudgeon with the HSI. The absence of clear relationships between the MTt/MTm-ratio, which can be considered as a measure for sensitivity could be due to other factors influencing those indicators. Possibly other sub-lethal endpoints such as growth or reproduction should be selected in order to assess the protective effect of MT on fish condition or health.

From this study we can conclude that different fish species exposed within a same pollution gradient differentially accumulate metals and differentially induce metal binding proteins. Perch accumulated highest levels of Cd and Zn but showed the lowest detoxification capacity. Only few and weak relationships between detoxification capacity and measured endpoints could be found. Probably more sensitive chronic effects have to be studied that are less affected by other environmental factors.
